# MLVA-16 typing of 295 marine mammal *Brucella *isolates from different animal and geographic origins identifies 7 major groups within *Brucella ceti *and *Brucella pinnipedialis*

**DOI:** 10.1186/1471-2180-9-145

**Published:** 2009-07-20

**Authors:** Marianne Maquart, Philippe Le Flèche, Geoffrey Foster, Morten Tryland, Françoise Ramisse, Berit Djønne, Sascha Al Dahouk, Isabelle Jacques, Heinrich Neubauer, Karl Walravens, Jacques Godfroid, Axel Cloeckaert, Gilles Vergnaud

**Affiliations:** 1 INRA, UR1282, Infectiologie Animale et Santé Publique, IASP, Nouzilly, F-37380, France; 2 Université Paris-Sud 11, CNRS, UMR8621, Institut de Génétique et Microbiologie, Orsay 91405, France; 3 Division of Analytical Microbiology, Centre d'Etudes du Bouchet, Vert le Petit, F-91710, France; 4 SAC Veterinary Services, Inverness, IV2 4JZ, UK; 5 Norwegian School of Veterinary Science, Department of Food Safety and Infection Biology, Section of Arctic Veterinary Medicine, Stakkevollveien 23, N-9010 Tromsø, Norway; 6 Norwegian National Veterinary Institute, Ullevålsveien 68, N-0106 Oslo, Norway; 7 Bundeswehr Institute of Microbiology, Department of Bacteriology, Neuherbergstr. 11, D-80937 Munich, Germany; 8 RWTH Aachen University, Department of Internal Medicine III, Pauwelsstraße 30, D-52074 Aachen, Germany; 9 Institut Universitaire de Technologie, Université François Rabelais, 29 rue du pont-volant, 37082 Tours cedex 2, France; 10 Friedrich Loeffler Institute, Institute of Bacterial Infections and Zoonoses, Naumburger Str. 96a, D-07743 Jena, Germany; 11 Veterinary and Agrochemical Research Centre (VAR), Groeselenberg 99, B-1180 Brussels, Belgium; 12 DGA/MRIS-Mission pour la Recherche et l'Innovation Scientifique, 7-9, rue des Mathurins, Bagneux F-92221, France

## Abstract

**Background:**

Since 1994, *Brucella *strains have been isolated from a wide range of marine mammals. They are currently recognized as two new *Brucella *species, *B. pinnipedialis *for the pinniped isolates and *B. ceti *for the cetacean isolates in agreement with host preference and specific phenotypic and molecular markers. In order to investigate the genetic relationships within the marine mammal *Brucella *isolates and with reference to terrestrial mammal *Brucella *isolates, we applied in this study the Multiple Loci VNTR (Variable Number of Tandem Repeats) Analysis (MLVA) approach. A previously published assay comprising 16 loci (MLVA-16) that has been shown to be highly relevant and efficient for typing and clustering *Brucella *strains from animal and human origin was used.

**Results:**

294 marine mammal *Brucella *strains collected in European waters from 173 animals and a human isolate from New Zealand presumably from marine origin were investigated by MLVA-16. Marine mammal *Brucella *isolates were shown to be different from the recognized terrestrial mammal *Brucella *species and biovars and corresponded to 3 major related groups, one specific of the *B. ceti *strains, one of the *B. pinnipedialis *strains and the last composed of the human isolate. In the *B. ceti *group, 3 subclusters were identified, distinguishing a cluster of dolphin, minke whale and porpoise isolates and two clusters mostly composed of dolphin isolates. These results were in accordance with published analyses using other phenotypic or molecular approaches, or different panels of VNTR loci. The *B. pinnipedialis *group could be similarly subdivided in 3 subclusters, one composed exclusively of isolates from hooded seals (*Cystophora cristata*) and the two others comprising other seal species isolates.

**Conclusion:**

The clustering analysis of a large collection of marine mammal *Brucella *isolates from European waters significantly strengthens the current view of the population structure of these two species, and their relative position with respect to the rest of the *Brucella *genus. MLVA-16 is confirmed as being a rapid, highly discriminatory and reproducible method to classify *Brucella *strains including the marine mammal isolates. The *Brucella2009 *MLVA-16 genotyping database available at http://mlva.u-psud.fr/ is providing a detailed coverage of all 9 currently recognized *Brucella *species.

## Background

*Brucellae *are Gram-negative, facultative, intracellular bacteria that can infect many species of animals and man. Six species were classically recognized within the genus *Brucella*: *B. abortus*, *B. melitensis*, *B. suis*, *B. ovis*, *B. canis*, and *B. neotomae *[1,2]. This classification is mainly based on differences in pathogenicity, host preference, and phenotypic characteristics [1-3]. Three additional species have been recently included in the genus *Brucella*, i.e. *B. ceti *and *B. pinnipedialis *isolated from marine mammals, with cetaceans (dolphin, porpoise, and whale species) and pinnipeds (various seal species) as preferred hosts respectively [4], and *B. microti *isolated from the common vole [5]. From a phenotypic point of view, *B. ceti *and *B. pinnipedialis *can be distinguished by their growth requirement for CO_2 _and their oxidative metabolism [6,7].

The phylogenetic significance of this separation is supported by molecular analyses. At the molecular level, evidence for two distinct marine mammal *Brucella *subpopulations subsequently given species rank and designated *B. ceti *and *B. pinnipedialis *has been initially provided by study of DNA polymorphism at the porin-encoding *omp2 *locus [8]. This was further confirmed by an infrequent restriction site-PCR (IRS-PCR) method, reflecting the higher number of IS*711 *elements in the genome of marine mammal isolates compared to terrestrial mammal *Brucella *species [9-11]. IRS-PCR revealed six specific DNA fragments useful for the detection and identification of marine mammal *Brucella *isolates and the presence of a putative genomic island only in seal isolates except for hooded seal isolates [11,12]. Interestingly to date three human cases, one from New Zealand and two from Peru, with *Brucella *infections presumably of marine origin, have been described according to the specific molecular markers cited above, and may point towards a zoonotic potential of these marine mammal *Brucella *species [13,14]. One human case with laboratory acquired infection has also been reported [15].

In the past few years, polymorphic tandem repeat loci have been identified by analysing published genome sequences of *B. melitensis *16 M, *B. suis *1330, and *B. abortus *9–941 [16-18]. Hundreds of *Brucella *strains have been typed to allow the development of an assay, called MLVA-16 assay (Multiple Locus VNTR Analysis) [5,17-23]. The sixteen loci have been grouped in 3 panels, called panel 1 (8 minisatellite loci), panel 2A (3 microsatellite loci) and panel 2B (5 microsatellite loci) [17,20]. Panel 1 has shown to be useful for species identification. Panel 2A and panel 2B increased the discriminatory power. Panel 2B was selected to contain the more highly variable markers, which is why this panel is often given a lower weight in clustering analysis [20,21]. Three of the five octamers in panel 2B have been initially evaluated by Bricker *et al*. [16]. The MLVA-16 assay provides a clustering of strains that is in accordance with the currently recognized *Brucella *species and biovars isolated from terrestrial mammals.

The aim of this study was to evaluate the MLVA-16 assay for the classification of marine mammal *Brucella *isolates, using 294 marine mammal *Brucella *strains obtained from 173 animals representing a wide range of marine mammal species from different European geographic origins (excluding the Mediterranean sea). This study also included the strain, presumably of marine origin, isolated from a patient in New Zealand [14]. A tentative overview of the global *Brucella *population structure was produced by comparison with published typing data.

## Results

All strains could be typed at all loci, with few exceptions for panel 2B loci. At the loci bruce04, bruce09 and bruce16, multiple bands were observed in the PCR products of 12, 9 and 6 strains, respectively. This may suggest that in some occasions multiple alleles are present in the DNA preparation. Besides, two strains were negative in PCR either for bruce07 or bruce30. In 69 animals, strains were initially isolated from different organs, contributing 121 extra strains. In sixteen among these animals, more than one genotype was observed (in one animal 5 different genotypes were found). In most cases, these genotypes were also observed in at least one other animal. In five cases, at least one of the genotypes was unique in the present collection, suggesting that the presence of multiple genotypes could be the result of a mutation event that occurred in the course of infection. Three of these new genotypes were the result of one repeat unit changes at a single locus. The other two were a 2 repeat units change in bruce04 and a four repeat units change in bruce09. These observations suggest that occasionally the most highly mutable loci may vary in the course of infection. They also do not exclude the possibility that animals carrying multiple variants may have been infected by multiple strains present within the community.

**Figure 1:**
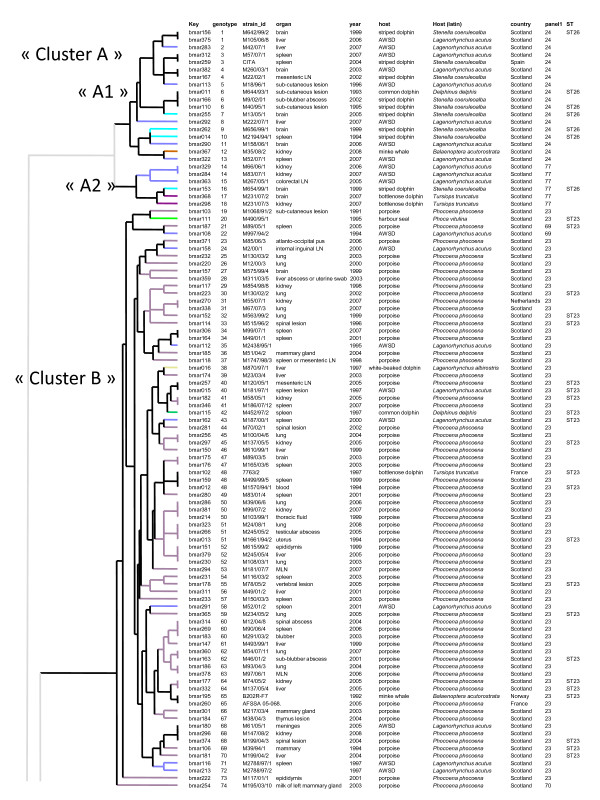
**MLVA-16 clustering analysis of 102 *B. ceti *strains defines three groups of strains**. All *B. ceti *isolates cluster into a first part (genotypes 1 to 74) of the dendogram constructed from MLVA-16 testing of 294 *Brucella *strains obtained from 173 marine mammals (pinnipeds, otter and cetaceans) and one human patient from New Zealand. One strain per genotype and per animal is included (consequently some animals are represented by more than one strain), 196 entries are listed corresponding to 117 genotypes. In the columns, the following data are presented: DNA batch (key), genotype, strain identification, organ, year of isolation, host (AWSD: Atlantic White Sided Dolphin), host (Latin name), geographic origin, MLVA panel 1 genotype, sequence type when described by Groussaud *et al*. [25]. The colour code reflects the host species (see Figure 3 for detailed correspondence). No colour was used when identical genotypes were observed in different host species. The letter nomenclature proposed by Groussaud *et al*. is used (*B. ceti*, cluster A (ST26) further subdivided into A1 and A2 and cluster B (ST23)).

**Figure 2:**
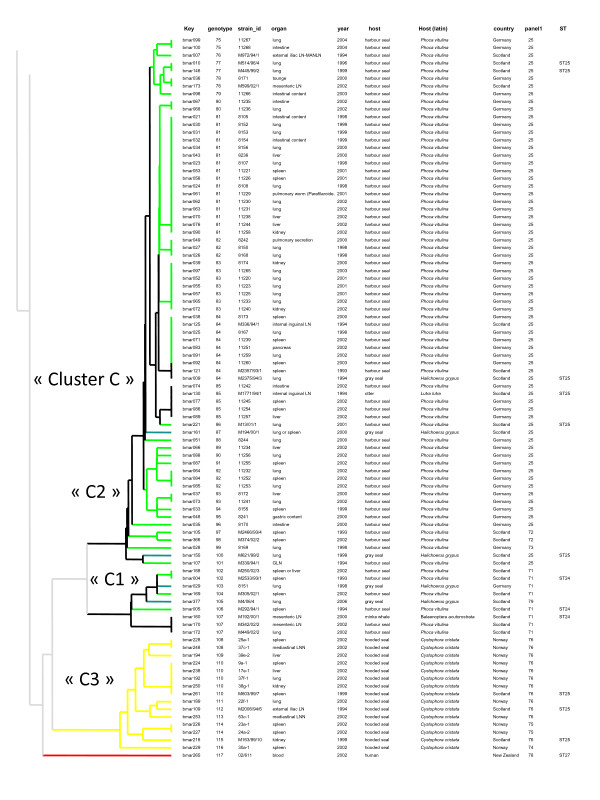
**MLVA-16 clustering analysis of 93 *B. pinnipedialis *strains defines 3 groups of strains**. All *B. pinnipedialis *isolates cluster together in the second part (genotypes 75 to 117) of the dendogram constructed from MLVA-16 testing of 294 *Brucella *isolates obtained from 173 marine mammals (pinnipeds, otter and cetaceans) and one human patient from New Zealand. In the columns, the following data are presented: DNA batch (key), genotype, strain identification, organ, year of isolation, host (AWSD: Atlantic White Sided Dolphin), host (Latin name), geographic origin, MLVA panel 1 genotype, sequence type when described by Groussaud *et al*. [25]. The colour code reflects the host species (see Figure 3 for detailed correspondence). No colour was used when identical genotypes were observed in different host species. The red branch (genotype 117) corresponds to the human isolate (ST27). The letter nomenclature proposed by Groussaud *et al*. is used (*B. pinnipedialis*, cluster C, including C1 (ST24), C2 (ST25) and C3 (ST25)).

**Figure 3:**
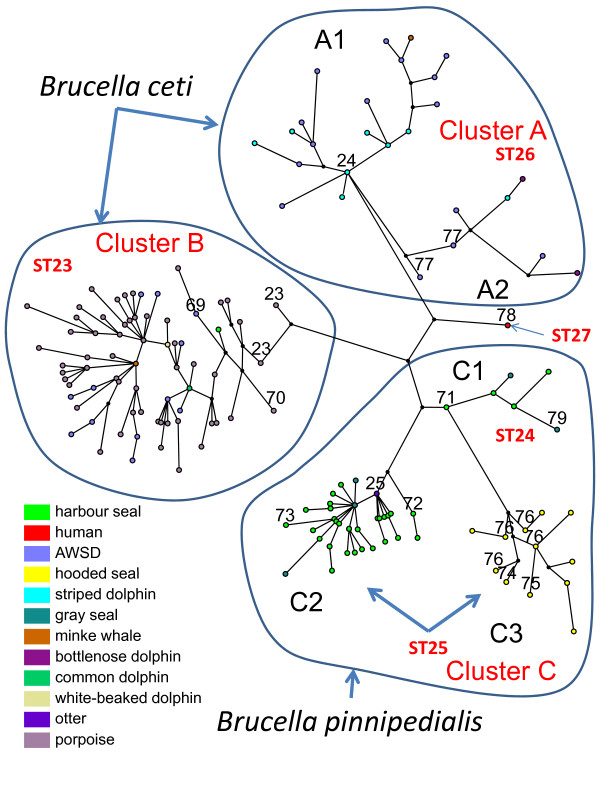
**Maximum parsimony analysis on 117 marine mammal *Brucella *genotypes**. Each coloured circle corresponds to one MLVA-16 genotype from a marine mammal species. Numbers in black (23, 24, 25, 69 to 79) indicate the MLVA the panel 1 genotype for the colour circle below. The panel 1 genotype along daughter branches is indicated only when it is different from the proposed parent node (i.e. in cluster A, all strains are panel 1 genotype 24 in subcluster A1 or 77 in subcluster A2). The tentative MLST sequence type (ST23 to ST27) as predicted from strains shared between this study and [25] is indicated, together with species assignment. The host species colour code indicated is the same as in Figures 1 and 2 (AWSD: Atlantic White Sided Dolphin).

**Figure 4:**
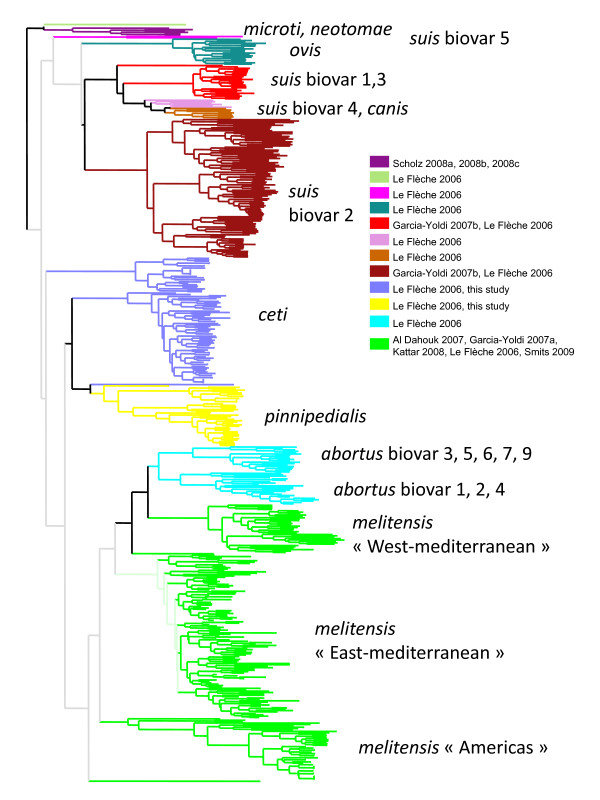
**Current view of the global population structure of the *Brucella *genus**. Clustering was done using the Neighbor Joining (NJ) algorithm. The microti/neotomae cluster was used to root the tree. The dendrogram is based upon more than 500 genotypes, observed by typing more than 750 strains [see Additional file 1]. The terrestrial mammal strains data were compiled from [5,17,19-23,37]. The colour code reflects the *Brucella *species (or some highly specific biovars). The publications from which the data were derived are indicated. The long blue branch close to the *B. pinnipedialis *cluster represents the human isolate from New Zealand (MLST ST27).

The 294 investigated marine mammal *Brucella *isolates which originated from 173 animals and one patient clustered in 117 different genotypes using the complete MLVA-16 assay. One representative for each genotype and animal was used for analysis, totalling 196 strains (Figures 1, 2, 3). Three main groups were identified, the *B. ceti *group, the *B. pinnipedialis *group and a third group comprising the human isolate from New Zealand. The 117 representative genotypes were compared with the 18 terrestrial mammal *Brucella *reference strains and published data (Figure 4). The 3 clusters were clearly separated from all the terrestrial mammal isolates.

The cetacean group composed of 102 strains presenting 74 genotypes (1–74) (Figure 1) could be separated into three major subclusters. The first two subclusters A1 and A2, from genotype 1 to 18 were only composed of dolphin isolates and one minke whale isolate (9 isolates from striped dolphins (*Stenella coeruleoalba*), 11 from Atlantic white sided dolphins (*Lagenorhynchus acutus*), 2 from bottlenose dolphins (*Tursiops truncatus*) and 1 from a common dolphin (*Delphinus delphis*)). These two subclusters correspond to sequence type ST26 [24], MLVA panel 1 genotype 24 (subcluster A1) and 77 (subcluster A2, Figure 1 and Figure 3), and together correspond to cluster A in [25] (Figure 3). The third subcluster, from genotype 19 to 74 corresponds to MLST sequence type 23, MLVA-16 panel 1 genotypes 23, 69 and 70, and is cluster B in [25] (Figure 1 and Figure 3). This subcluster was composed of 78 strains. Sixty-four were obtained from porpoises, 12 from 4 species of dolphins (9 from Atlantic white sided dolphin (*Lagenorhynchus acutus*), one from a white-beaked dolphin (*Lagenorhynchus albirostris*), one from a bottlenose dolphin (*Tursiops truncatus*), one from a common dolphin (*Delphinus delphis*), and one from a minke whale (*Balaenoptera acutorostrata*) isolated in Norway in 1995 [10] (Figure 1). An exception was the bmar111 (strain number M490/95/1), with the genotype 20, isolated in Scotland from a harbour (or common) seal (*Phoca vitulina*) and which belongs to the *B. ceti *group (Figure 1). This is, however, in agreement with previous observations, either phenotypic [26] or molecular, including MLVA typing [25]. This particular strain carries the two specific IRS-PCR fragments (II and III) of the *B. ceti *strains [11], and the PCR-RFLP pattern of the *omp2 *genes is similar to that of *Brucella *strains isolated from porpoises [8].

The 93 representative *B. pinnipedialis *strains presented 42 different genotypes (75–116) (Figure 2) corresponding to cluster C in [25]. This group of isolates could similarly be further divided in three major subclusters. The first subcluster (genotype 75 to 101) was composed of several seal isolates (harbour seal and grey seal (*Halichoerus grypus*)) and the isolate from a European sea otter (*Lutra lutra*). It corresponds to MLST sequence type 25, MLVA panel 1 genotypes 25, 72, 73, and cluster C2 in [25]. The second subcluster (MLVA genotypes 102 to 107) corresponds to MLST sequence type 24, MLVA panel 1 genotypes 71 and 79 and is cluster C1 in [25]. Interestingly, the hooded seal isolates (15 strains) were exclusively clustered in 9 closely related genotypes, forming the third subcluster of the pinniped isolates (genotype 108 to 116) called C3 in [25]. Most of the hooded seal isolates analysed in this study were isolated in Norway in 2002 [27] and there were also 4 hooded seal isolates from Scotland that clustered with the Norwegian isolates. One of the 93 strains of the *B. pinnipedialis *group was obtained from a cetacean. This strain (M192/00/1), identified as bmar160 with the genotype107 in Figure 2, was isolated from a minke whale in Scotland in 2000. This strain was also demonstrated as a *B. pinnipedialis *strain by other molecular markers, as described by Maquart *et al*. [12] and Groussaud *et al*. [25].

The human isolate 02/611 (bmar265) from New Zealand (genotype 117 (Figure 2)), clearly separates from the clusters described above but fits in an intermediate position within the marine mammal groups of isolates in agreement with previous MLST analyses [28]. Figure 3 suggests a position closer to the *B. ceti *group in agreement with the phenotypic behaviour [14], but the typing of more strains from Pacific waters [29-31] will be needed in order to achieve a more conclusive cluster analysis.

Owing to the inclusion of 40 representative strains in duplicate, the results described above could be compared to those recently described by Groussaud *et al*. who studied 74 marine mammal isolates by multilocus sequence typing, multilocus sequence analysis (MLST, MLSA) and MLVA. Duplicate typing was useful since Groussaud *et al*. used a partially different set of 21 VNTRs [25] (9 loci are common, including three loci from panel 1 (Bruce 08, 45, 55), one from panel 2A (Bruce18) and the whole panel 2B).

## Discussion

Since 1994, marine mammal *Brucella *strains have been isolated and characterized, both phenotypically and by means of different molecular typing methods. This led to the division of the marine mammal *Brucella *strains in 2 species i.e. *B. ceti *on one side and *B. pinnipedialis *on the other side defined by oxidative metabolism patterns and CO_2 _requirement for growth, and a number of subclusters defined by complementary molecular analysis methods.

This MLVA-16 study is, to date, the most important one in terms of number of strains analysed and number of animal species from which these strains have been isolated. These strains were isolated from animals stranded, caught or killed for scientific purposes in the waters surrounding Europe, from the Barents Sea, above the Arctic Circle to the Atlantic coast of Spain. For the 295 strains analysed, using the MLVA-16 assay, 117 genotypes were resolved and seven clusters were identified, (i) two clusters almost exclusively composed of dolphin isolates, (ii) the predominantly porpoise cluster of strains (which also includes several strains isolated from dolphins), (iii) two main seal species clusters, (iv) the hooded seal cluster, and (v) the human isolate. The last cluster might correspond to Pacific Ocean isolates [29-31], which are underrepresented in the present collection. The hooded seal cluster of strains was composed of strains from Scotland and Norway. The low level of genetic diversity between the hooded seal isolates from Scotland and from Norway could indicate that all the investigated hooded seals originated from the same population of animals. The population that was sampled between Svalbard and Greenland have their breeding area in the pack ice north of Jan Mayen (West Ice), but except for the few weeks on ice during birth, mating and moulting, the hooded seal is a typical pelagic and a migratory species with a huge geographical range [27]. It is thus likely that the hooded seals stranded in Scotland were originally from this area rather than from the more western bound populations of hooded seals that breed in the Davis Strait and at the east coast of Canada.

Groussaud *et al*. [25] analysed the diversity of marine mammal isolates by MLVA using another selection of VNTRs, including all 8 loci defining the HOOF-prints MLVA assay described by Bricker *et al*. [16] and 13 additional loci characterised by Le Flèche *et al*. [17] and Whatmore *et al*. [18]. This panel of 21 VNTR loci corresponded to a 21-locus MLVA scheme sharing 9 loci with MLVA-16 and also provides a high degree of diversity. In this previous study, multilocus sequence types (STs) were determined, allowing the clustering of marine mammal isolates in five groups labelled ST23 to ST27. The closely related ST24 and ST25 were composed of the pinniped isolates, forming the cluster C. The hooded seal isolates define subcluster C3. ST26 was exclusively composed of dolphin isolates and formed the cluster A. The other cetacean isolates all clustered in the cluster B (ST23) and consisted of strains isolated from porpoises and dolphins. ST27 was represented by only one isolate from an aborted bottlenose dolphin foetus originating from the Western coast of the United States (strain F5/99) [28]. Our results are thus in excellent accordance with those published by Groussaud *et al*. [25] showing that the previously identified population structure of marine mammal *Brucella *strains is not significantly modified by the inclusion of a large number of strains from European waters. MLVA-16 results are also in accordance with the recently reported genomic structures of 24 marine mammal *Brucella *isolates for which three subgroups were identified [32]. In that study, one separate group was identified for the *B. pinnipedialis *strains, another subgroup included dolphin isolates and a third subgroup comprised dolphin and porpoise isolates. The only hooded seal isolate analysed in that study clustered in the *B. pinnipedialis *group but revealed a separate pattern with a 62 kb missing fragment, specific for this group and relevant for a distinct genetic background [32].

MLVA-16 classification in the present report revealed some exceptions like the M490/95/1 strain, isolated from a common seal, which was clustered in the *B. ceti *group of strains. This exception suggests that transmission from cetaceans to pinnipeds may occur. Although the currently recognized terrestrial mammal *Brucella *species also have a preferred host, they can be isolated from different hosts in regions where brucellosis is endemic, e.g. *B. melitensis *which has been isolated from cattle in the southern part of France [33].

The human isolate from New Zealand formed a separate seventh MLVA-16 cluster. Whatmore *et al*. [28] have shown that the F5/99 strain, isolated from an aborted bottlenose dolphin fetus from the Western coast of the United States (together with three human isolates, one from New Zealand and two from Peru) shared the same MLST genotype (ST27). Interestingly, at the *omp2 *level both strains were identical and also identical to isolates from the North Pacific minke whales. In all these strains the porin *omp2 *genes were different from those from marine mammal strains isolated on European coasts [30]. Briefly, the *omp2 *genes of these isolates from the Pacific share common features with both marine mammal (from Europe) and terrestrial mammal strains [29]. Another interesting observation is that all the Pacific isolates investigated so far (including the three reported human cases) carry fragment I identified by IRS-PCR which is part of a putative genomic island specific for *B. pinnipedialis *[12]. Since these cetacean isolates are quite distinct from European marine mammal isolates there might be a third marine mammal *Brucella *species or subspecies found in Pacific waters. Owing to the simplicity of MLVA-16 typing, and in particular of panel 1 which can be typed on regular agarose gels and already provides a high informativity in classifying marine mammal strains (Figure 3), more typing information on Pacific Ocean strains (including the strains described in [29-31]) will likely be made available in a near future. The *Brucella*2009 genotyping database available at http://mlva.u-psud.fr/ and based upon the data provided in Additional file 1 can be used for this purpose. Figure 4 shows the global population structure of the nine species currently constituting the *Brucella *genus, as can be revealed by MLVA-16 typing using this dataset (the extended data set provided here may provide new opportunities to evaluate additional methods for *Brucella *MLVA data clustering recently proposed [34]).

## Conclusion

MLVA-16 proved to be useful for molecular classification of a high number of marine mammal *Brucella *strains and allows the typing of large populations, while providing a clustering in agreement with all previously reported methods, together with a much higher discriminatory power. From the clustering achieved, a few representative strains can be selected for whole genome sequencing.

## Methods

### *Brucella *strains

MLVA analysis was performed on 294 isolates from 173 marine mammals and one human patient. The strains essentially originate from the Northern Atlantic, from three main sources, Scotland (216 isolates from 116 animals), Germany (58 isolates from 42 animals) [35] and Norway (18 isolates from 13 animals) [27]. Six additional strains from various geographic origins were analysed. Two strains were obtained from France (one strain from a bottlenose dolphin (*Tursiops truncatus*) and one from a harbour porpoise (*Phocoena phocoena*)), one from Spain (from a striped dolphin (*Stenella coeruleoalba*)) [36] and two from The Netherlands (two strains from one harbour porpoise (*Phocoena phocoena*)). The sixth strain was a human isolate from New-Zealand (strain 02/611 genotype 117) [14]. Strains (one strain per genotype and animal) are listed in Figures 1 and 2 and in Additional file 1.

### MLVA analysis

The method for selecting appropriate VNTRs has been described previously by Le Flèche *et al*. [17], adapted by Al Dahouk *et al*. (the initial MLVA-15 assay was completed by bruce19) [20]. The results were compared with the MLVA-16 results obtained for the 18 terrestrial mammal *Brucella *reference strains published previously by Le Flèche *et al*. [17] and additional published data [5,19-23,37]. The sixteen loci have been classified in 3 panels, called panel 1 (8 minisatellite loci), panel 2A (3 microsatellite loci) and panel 2B (5 microsatellite loci) [20]. Panel 1 was composed of bruce06, bruce08, bruce11, bruce12, bruce42, bruce43, bruce45, bruce55, useful for species identification. Panel 2, showing a higher discriminatory power, was split into two groups, panel 2A and 2B, composed of three (bruce18, bruce19, bruce21) and five (bruce04, bruce07, bruce09, bruce16, bruce30) markers, respectively. Panel 2B contains the more variable loci, and this panel can be given a lower weight in clustering analysis, as described by Al Dahouk *et al*. [20] and Kattar *et al*. [21].

### PCR amplification

*Brucella *DNA was prepared as previously described by Cloeckaert *et al*. [38]. PCR amplification was performed in a total volume of 15 μl containing 1 ng of DNA, 1× PCR reaction buffer, 1 U of Taq DNA polymerase (QBiogen, Illkirch, France), 200 μM of each deoxynucleotide triphosphate, and 0.3 μM of each flanking primer as described by Le Flèche *et al*. [17].

Amplifications were performed in a MJ Research PTC200 thermocycler. An initial denaturation step at 96°C for 5 minutes was followed by 30 cycles of denaturation at 96°C for 30 s, primer annealing at 60°C for 30 s, and elongation at 70°C for 1 min. The final extension step was performed at 70°C for 5 min.

Two to five microliters of the amplification product were loaded on a 3% standard agarose gel for analyzing tandem repeats with a unit length shorter than 10 bp (panel 2) and on a 2% standard agarose gel for all others (panel 1), and run under a voltage of 8 V/cm until the bromophenol blue dye had reached the 20 cm position. Gels were stained with ethidium bromide, visualized under UV light, and photographed (Vilber Lourmat, Marnes-la-Vallée, France). A 100-bp and a 20-bp ladder (EZ load 100 bp or 20 bp PCR Molecular Ruler, Biorad, Marnes-la-Coquette, France) were used as molecular size markers depending on the tandem repeat unit length. Gel images were managed using the BioNumerics software package (version 6.0, Applied-Maths, Belgium).

### Data analysis

Band size estimates were converted to a number of units within a character dataset using the BioNumerics software and the previously published allele calling convention [17]. Clustering analyses used the categorical coefficient and the UPGMA (unweighted pair group method using arithmetic averages) or Neighbor Joining algorithm. The use of categorical parameter implies that the character states are considered unordered. The same weight is given to a large or a small number of differences in the number of repeats at each locus. Maximum parsimony was done using BioNumerics, running 200 bootstrap simulations treating the data as categorical and giving the same weight to all loci.

## Authors' contributions

JG and GV coordinated contributions by the different participants. IJ, MT, GF, BD, SAD, HN, FR, KW and JG isolated and/or maintained strains and/or produced DNA. PLF did the MLVA genotyping work. GV and PLF were in charge of the BioNumerics database, error checking, clustering analyses. MM, AC and GV wrote the report. IJ helped to draft the manuscript. All authors read, commented and approved the final manuscript.

## Supplementary Material

Additional file 1 **MLVA-16 data**. The repeat copy numbers at each locus are indicated for each strain. Click here for file
